# Reconstruction and repair, using mini-plate and bone graft for persons living with HIV with giant cell tumor of long bone: retrospective analysis of a single-center experience

**DOI:** 10.1186/s12981-021-00406-3

**Published:** 2021-11-02

**Authors:** Biao Xu, Rui Ma, Wen-sheng Zhang, Qiang Zhang, Chang-song Zhao, Jie Wang

**Affiliations:** Department of Orthopedics, Beijing Ditan Hospital, Capital Medical University, No.8, Jingshun East Street, Chaoyang District, Beijing, 100015 China

**Keywords:** HIV, Giant cell tumor, Mini-plate, Bone morphologic repairment and reconstruction, Autogenous and allogeneic bone grafts

## Abstract

**Background:**

To evaluate the effect of reconstruction and repair, using a mini-plate and bone graft for HIV -positive patients with giant cell tumor of long bone.

**Methods:**

We conducted a retrospective analysis of 12 HIV positive patients with giant cell tumor of long bone. A non-HIV-positive cohort of patients, matched for age, sex, and disease type, was selected as the control group. From June 2012 to August 2020, curettage by ultrasonic scalpel was performed in all patients, combined with min- plate and bone graft treatment. All patients were followed- up for 18 to 60 months. Limb function was evaluated, using the MSTS93 scoring system, and any examples of postoperative recurrence, distant metastasis, complications, MSTS93 score, and fracture prognosis were recorded.

**Results:**

The mean age of HIV group was 43.5 years. The ratio of men to women was 11: 1. In all cases the histopathological diagnosis was clear, except the patients with primary malignant giant cell tumor of bone, including five, three, two, and two cases in the proximal tibia, distal femur, distal tibia, and talus, respectively. Following their surgery, all patients were followed up with an average of 31.24 ± 11.84 months. No local recurrence or pulmonary metastases were observed. Post-surgery, all the 12 patients showed good bone morphologic repair and reconstruction, good bone healing, good joint function, and no pathological fractures around their lesion. In the HIV group, one case of giant cell tumor in the proximal tibia showed mild articular surface collapse and mild valgus deformity of the knee joint but retained good joint function. The MSTS scores of excellent or good in the two groups comprised 83.3%, thus, there was no significant difference between them (P > 0.05). Compared with preoperatively, the MSTS scores in the HIV group were significantly improved, ranging from 7 to 11 points preoperatively to 24 to 27 points postoperatively; this difference was statistically significant (P < 0.05).

**Conclusion:**

Reconstruction and repair, using a mini-plate and bone graft for HIV -positive patients with giant cell tumor of long bone can achieve satisfactory results. The mini- plate requires little space and is flexible during reconstruction and fixation, significantly reducing complications such as surgical site infection, as well as preserving joint function and avoiding amputation; therefore, it is a safe and effective treatment method.

## Background

Giant cell tumor of the bone (GCT) is a primary bone tumor, which accounts for which accounts for between 5 and 7% of all primary bone tumors and 20% of all benign bone tumors [1].In China, its incidence is between 14 and 20%, which is higher than the 5% to 8% seen in other Eastern countries [2]. GCT has unpredictable biological behavior with potential invasion, local recurrence, and a low probability of distant metastasis [3–5], which tends to occur in people aged 20 to 40 years; the incidence is slightly higher in females than males. GCT often occurs in the meta-epiphyseal area of the limbs, especially in the distal femur and proximal tibia. GCT grows in an expansive manner and can easily penetrate the cortex of the bone and even cause pathological fracture. According to reports in the literature, the incidence of such pathological fractures is between 9 and 30% [6–10].

GCT is a type of osteolytic tumor, with mononuclear cells and osteoclasts multiple nuclear cells comprising the main components. HIV infection leads to a decrease in the CD4 T cell count and dysfunction of macrophages and monocytes, ultimately leading to immunodeficiency [11, 12]. The question we are interested in is: How are these patients treated with surgical intervention? However, at present, few literatures reported about HIV positive patients with GCT with pathological fracture and there are no effective treatments of this disease currently.

## Methods

This study was reviewed and approved by the ethics committee of the Department of Orthopedics, Beijing Ditan Hospital, Capital Medical University, and was conducted in accordance with the Declaration of Helsinki and its subsequent amendments. Written consent was obtained from all participants prior to the start of the study. From June 2012 to August 2020, 12 HIV-positive patients with giant cell tumor in their limbs were selected from the Beijing Ditan Hospital Medical Center for Surgical Treatment. All patients were followed up for 18 to 60 months following their surgery. HIV infection was confirmed in all patients by enzyme-linked immunosorbent assay (ELISA), with the lower limit of RNA detection < 20copies/ml.

The blood CD4 T cell count, HIV-RNA viral load, and the administration of the patients’ highly active antiviral therapy (HAART) regimens were collected from all patients during the perioperative period (Table 1). All tumors were treated with expanded intracapsular curettage. During the curettage process, the bone wall was ground and drilled using an ultrasonic scalpel at high speed. The tumor cavity was inactivated with 10% hypertonic saline.

**Table 1:** Demographic

Case	Sex	Age	Site	Follow-up	Pre-MSTS	Post-MSTS
1	M	16	Proximal tibia	18	7	26
2	M	33	Distal femur	24	9	27
3	M	56	Proximal tibia	20	10	24
4	M	48	Distal femur	32	8	25
5	M	44	Distal tibia	34	9	27
6	F	65	Proximal tibia	36	11	26
7	M	36	Distal tibia	40	10	24
8	M	68	Proximal tibia	42	7	26
9	M	52	Talus	46	9	25
10	M	21	Proximal tibia	48	11	27
11	M	55	Talus	52	8	25
12	M	28	Distal femur	60	10	24

**Fig. 1:**
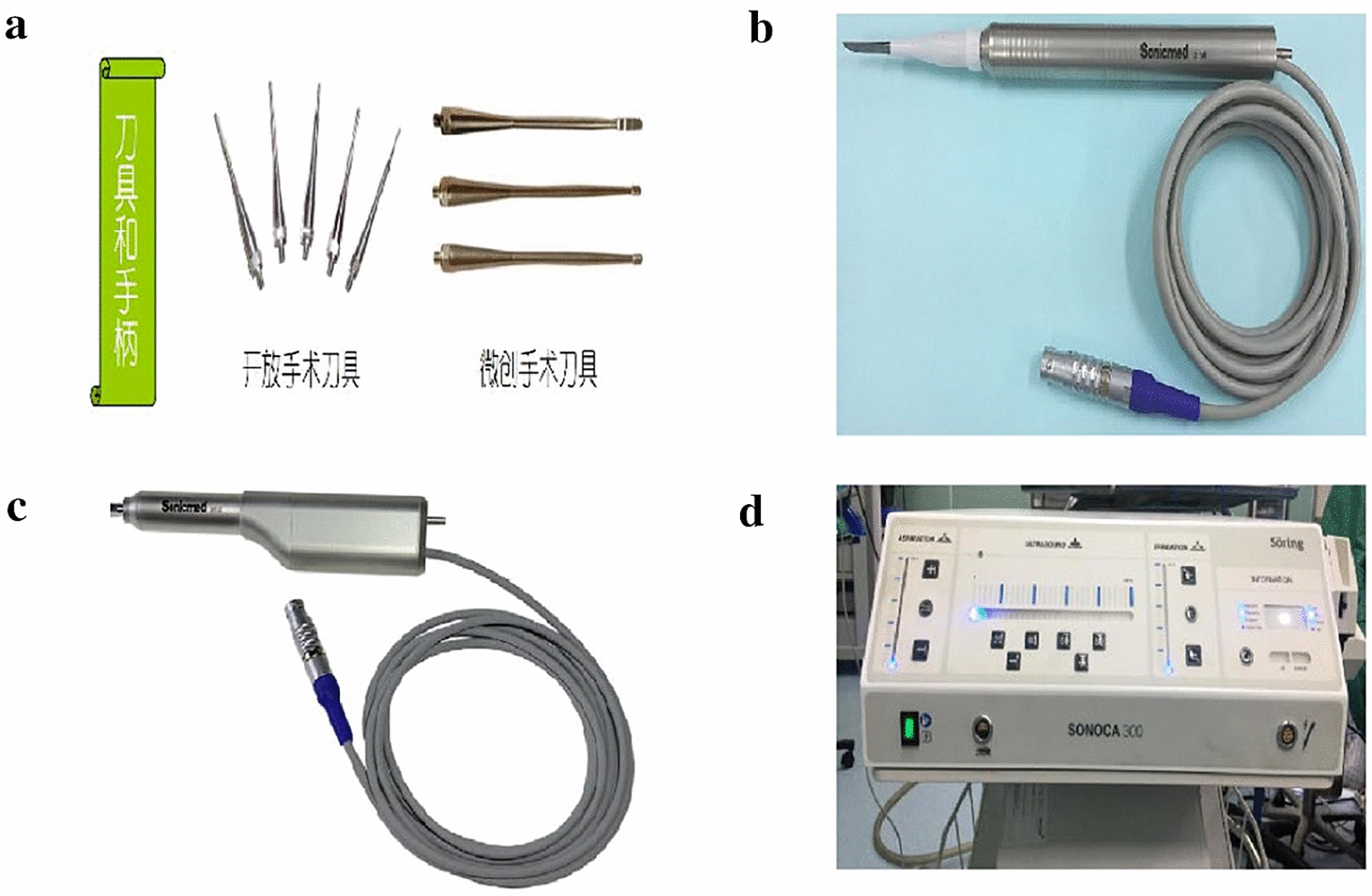
SONOCA 300 SoringGmbH: Surgical tools (**a**),Cut the bone handle (**b**),Grind bone handle (**c**), main engine and cooling system (**d**)

An ultrasonic treatment system developed by SONOCA 300 SoringGmbH comprises three parts: the main engine, the handle and cutting tool, and the cooling system (Fig. 1). A computer controls the electrical signals of the ultrasonic frequency signal generator, a power amplifier amplification drive after ultrasonic transducer, an ultrasonic transducer produces vibrations under the action of electrical signals, the amplitude will drives the cutting tool following amplitude amplification, working frequency of 40 ± 2 kHz, the implementation of automatic frequency tracking, equipped with 3 mm and 2 mm belt teeth and groove cutter, cut to fit different needs. The ultrasonic energy output of the cutter is set at 30%. The handle has a self-cooling system, and the cutter can rotate slowly clockwise or counterclockwise, as well as alternate positive and negative rotation, to increase the cutting ability.

**Fig. 2:**
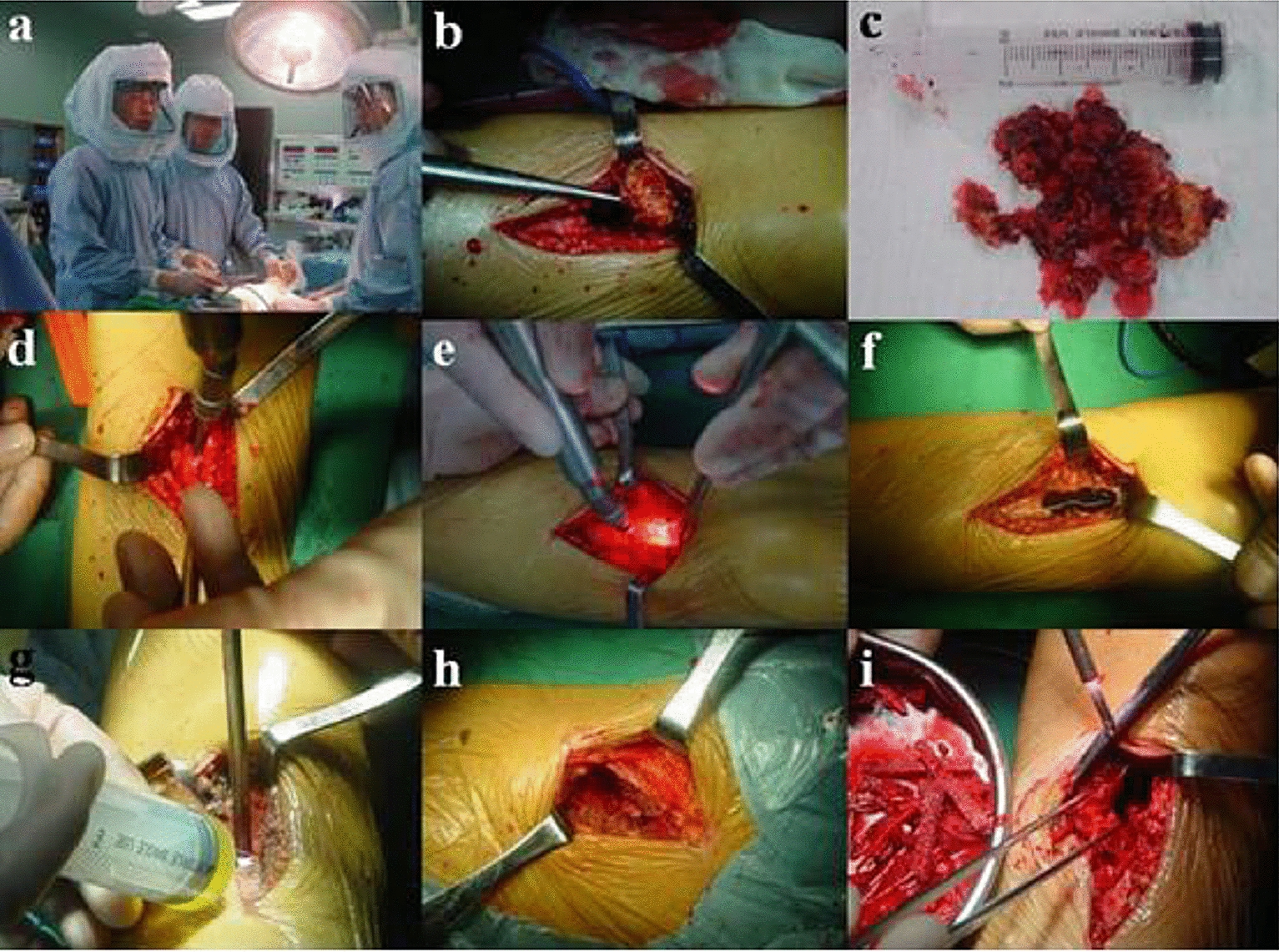
During the operation, the lesion was scraped and the bone cavity was polished with ultrasonic knife (**a**–**f**), the polished inner wall of the bone cavity was burned with anhydrous alcohol, and local methotrexate chemotherapy was performed (**g**), Autogenous iliac crest or allograft (**h**, **i**)

All patients’ tumors were first scraped with a spoon, the tumor wall was ground using the ultrasonic scalpel, and then local chemotherapy with methotrexate and a utogeous bone graft were used for treatment. The window was fully open when shaving, grinding carefully and repeatedly scratched tumor cavity wall and cavity bone crest, so the naked eye could see no tumor bone wall, reserved was not pay attention to keep the normal bone tumor invasion and epiphyseal plate, saline flushing repeatedly, local chemotherapy using methotrexate gelatin sponge, measuring the bone cavity size, iliac bone graft taken from the body cavity filling. Mini-plate fixation screws suitable for the anatomical location in question were selected for internal fixation (Fig. 2).

In all patients, general radiography, chest X-ray, computed tomography (CT), and/or magnetic resonance imaging (MRI) were performed, on more than one plane. In addition, all patients underwent needle puncture cytology and/or an open biopsy. The Musculoskeletal Tumor Society (MSTS) 93 score was used to evaluate the postoperative condition of limbs [13]. The upper limb function score comprised six items, including limb pain, activity function, acceptability, hand position, dexterity, and lifting ability. The lower extremity function score also comprised six items: limb pain, motor function, acceptability, support use, walking function, and gait.

## Statistical analysis

Sex, age, site of occurrence, surgical treatment, postoperative complications, limb function, and HIV-related clinical data were collected for all patients and statistically analyzed. SPSS 18.0 software was used to perform the statistical analyses. Measurement data were expressed as means ± s, and a P-value < 0.05 was considered statistically significant.

## Results

The mean age of HIV group was 43.5 years. The ratio of men to women was 11: 1. In all cases the histopathological diagnosis was clear, except the patients with primary malignant giant cell tumor of bone, including five, three, two, and two cases in the proximal tibia, distal femur, distal tibia, and talus, respectively (Table 2).

**Table 2:** HIV-related details

Case	Type of ART	Duration of HIV (year)	Duration of treatment	CD4 nadir	HIV viral load at diagnosis	CD4 at time of surgery	HIV viral load at time of surgery
1	3TC + TDF + NVP	2	2	334	5800	467	423
2	3TC + TDF + NVP	3	3	312	649,674	332	207
3	3TC + TDF + DTG	1	1	445	11,878	601	TND
4	3TC + TDF + NVP	2	2	281	28,259	380	4282
5	RAL + FTC/TDF	3	3	211	17,048	246	8507
6	3TC + TDF + NVP	2	2	327	4106	476	TND
7	3TC + TDF + LPV	3	3	470	8762	587	< 20
8	3TC + TDF + EFV	2	2	268	1108	386	94
9	3TC + TDF + NVP	4	4	276	28,430	337	2553
10	3TC + AZT + EFV	2	2	403	43,016	567	29,033
11	3TC + TDF + NVP	3	3	369	9149	465	TND
12	3TC + TDF + NVP	2	2	252	30,805	375	TND

Following their surgery, all patients were followed up with an average of 31.24 ± 11.84 months. No local recurrence or pulmonary metastases were observed. All patients showed good morphologic repair and reconstruction of bone, good bone healing, good joint function, and no pathological fractures around the lesion. In the HIV group, one case of giant cell tumor of proximal tibia showed mild articular surface collapse and mild valgus deformity of the knee joint, but still exhibited good joint function. The MSTS scores of excellent and good in the two groups was 83.3%, and thus there was no statistically significant difference (P > 0.05) (Table 3). Compared with the preoperative scores, the postoperative MSTS scores of the HIV group were significantly improved, ranging from 7 to 11 points preoperatively to 24 to 27 points postoperatively (P < 0.05) (Table 2).

**Table 3:** Comparison of excellent and good rates of postoperative MSTS scores between the two groups [cases (%)]

Group	Number	Poor	Medium	Good	Well	Nice
HIV group	12	0 (0)	2 (16.7)	4 (33.3)	6 (50)	8 (83.3)
Control group	12	0 (0)	2 (16.7)	3 (25)	7 (58.3)	11 (83.3)

**Fig. 3:**
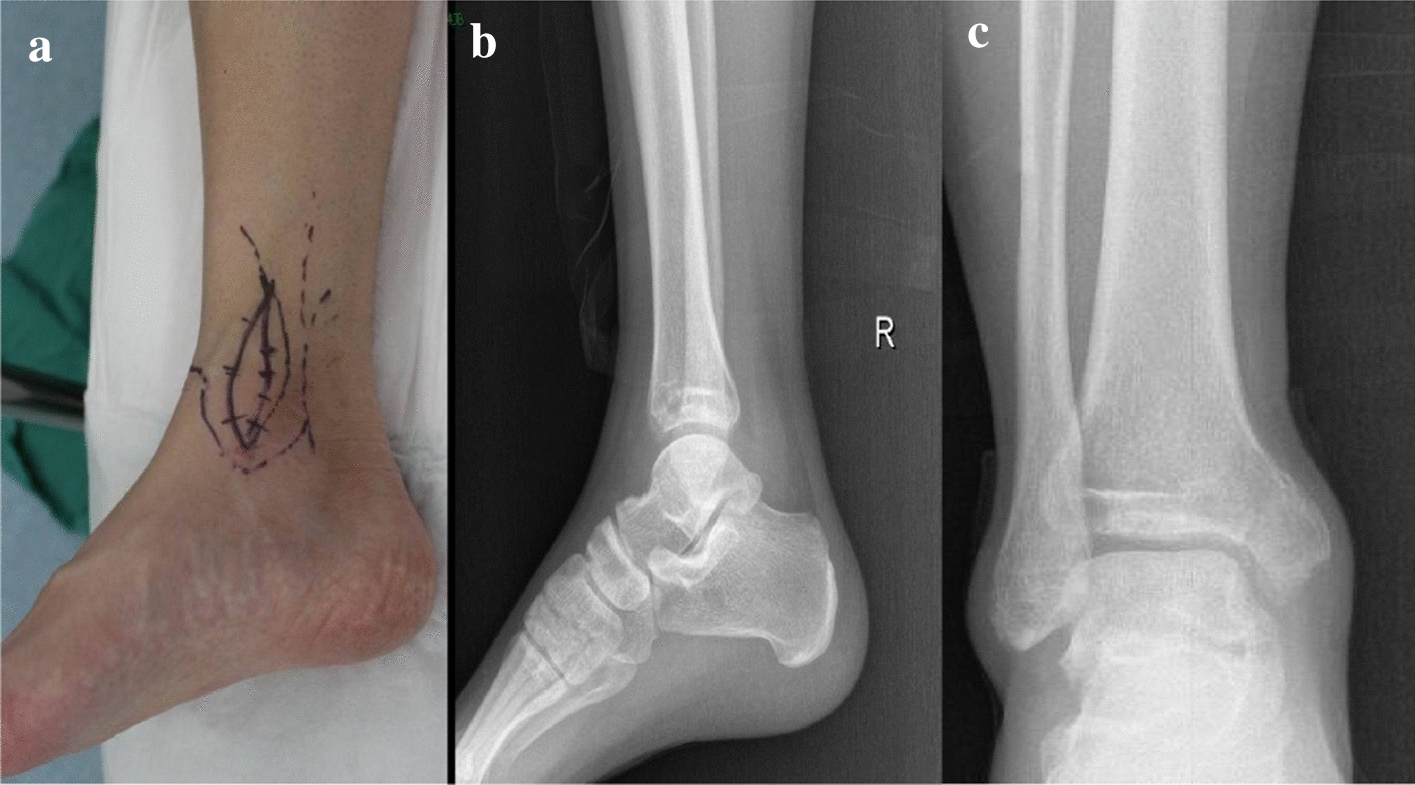
Preoperative general image (**a**), X—ray of the ankle joint showed a rounded, cystic destruction of the distal right tibia with expansion and thinning of the bone cortex (**b**, **c**)

**Fig. 4:**
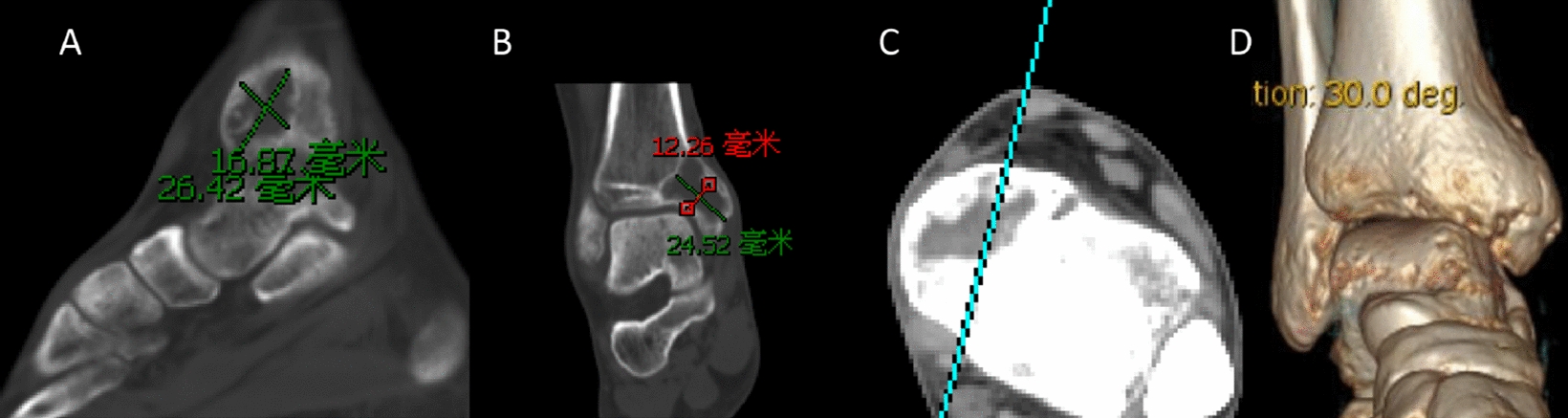
CT results showed that the right tibia distal round cystic bone destruction, expansions, internal trabeculae disruption, bone cortical rupture Swelling of the surrounding soft tissue (**a**–**d**)

**Fig. 5:**
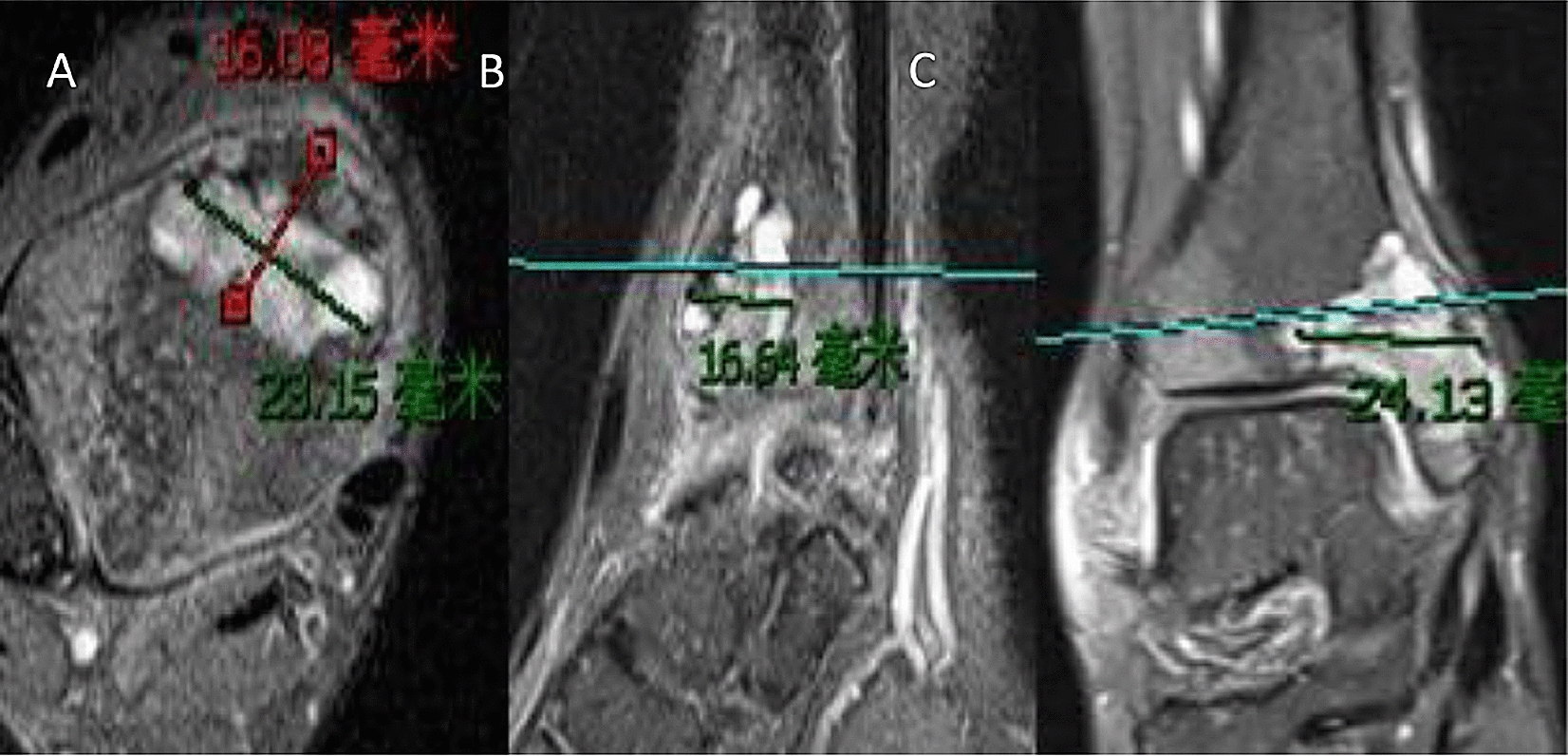
MRI plain scan of the ankle joint (October 20, 2017): a mass at the distal end of the right tibia, about 29 mm × 13 mm × 24 mm abnormal Constant signal, TIWL shows equisignal with clear margin and visible bone destruction, which is considered as giant cell tumor of bone (**a**–**c**)

**Fig. 6:**
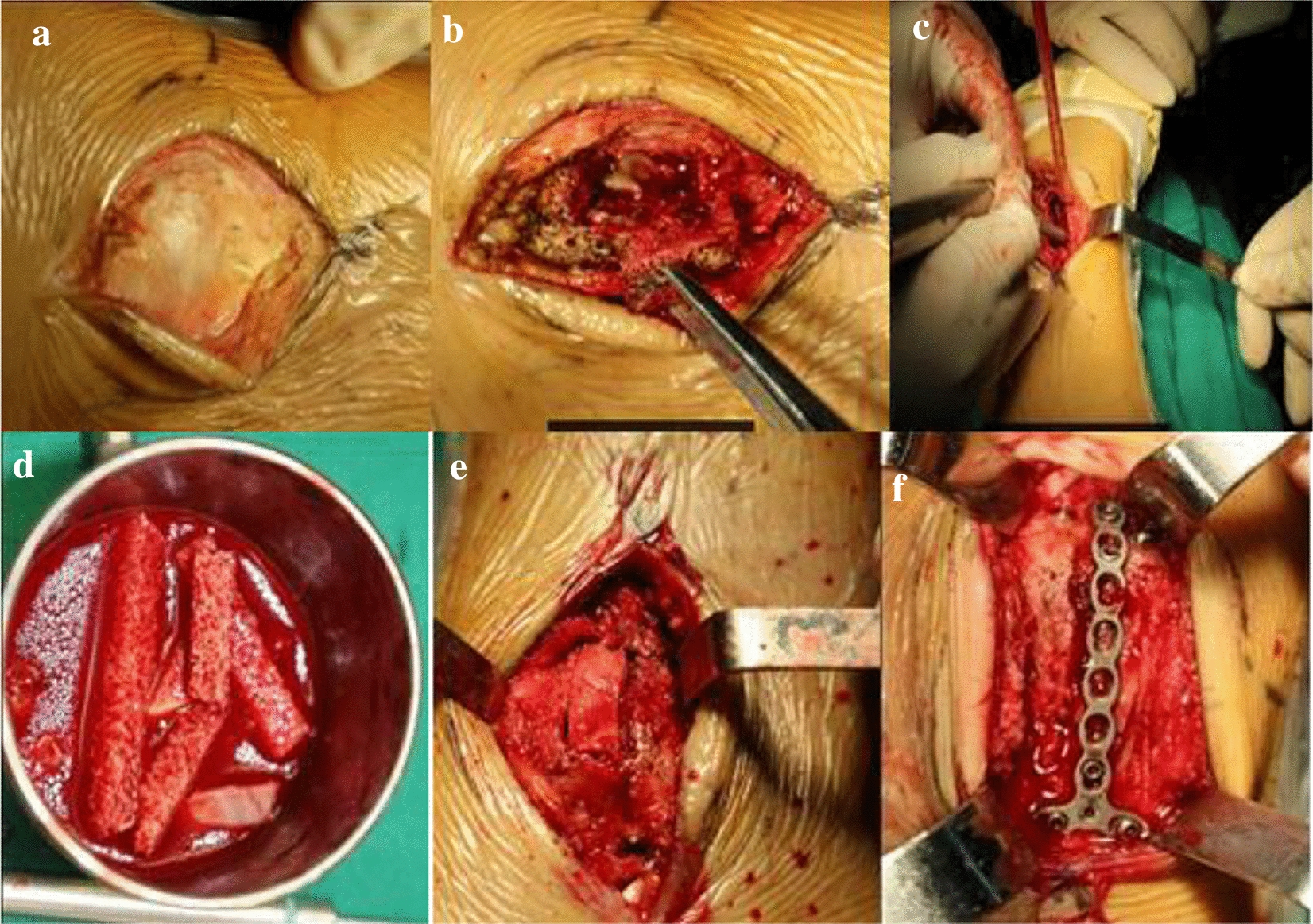
Tumor curettage with ultrasonic knife, 95% alcohol, local chemotherapy with methotrexate (**a**–**c**), bone graft fusion (**d**, **e**)and internal fixation with micro plate and screw (**f**) were performed

**Fig. 7:**
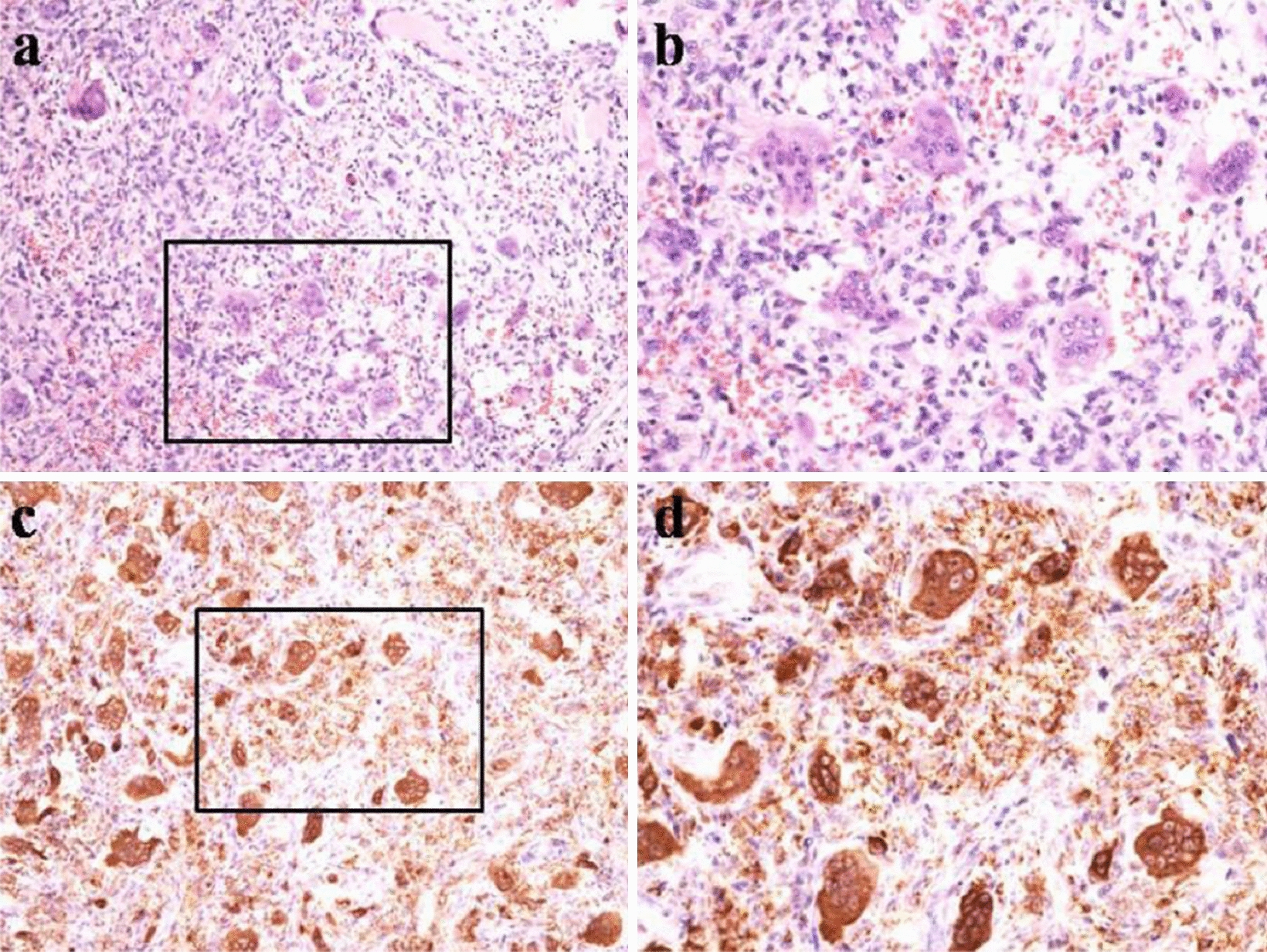
HE staining (×20, ×40): A large number of giant cells and stromal cells were observed, accompanied by a small amount of focal hemorrhage and fibrous tissue hyperplasia(**a**, **b**), Immunohistochemical staining (20×, 40×): CD68 antigen positive (**c**, **d**)

**Fig. 8:**
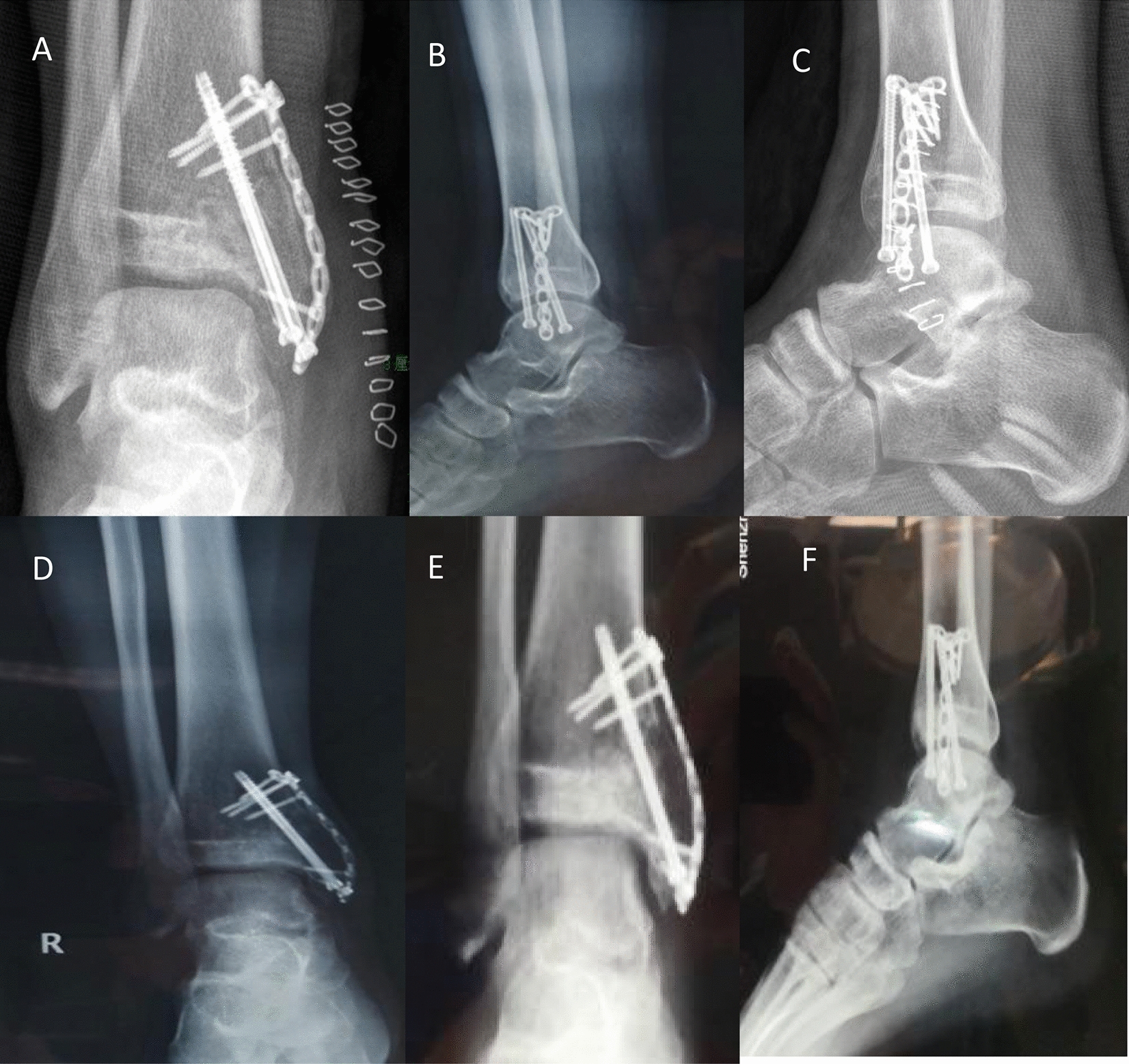
Postoperative anteroposterior and lateral X-ray examination of the ankle joint at 1 week (**a**, **b**), 1 year (**c**, **d**) and 2 years (**e**, **f**): postoperative resection of the distal right tibia lesion with bone graft fusion plate Postoperative screw fixation. The bone graft was filled and fused well. Steel plate and screw fixed firmly, no fracture, bone nonunion, etc

**Fig. 9:**
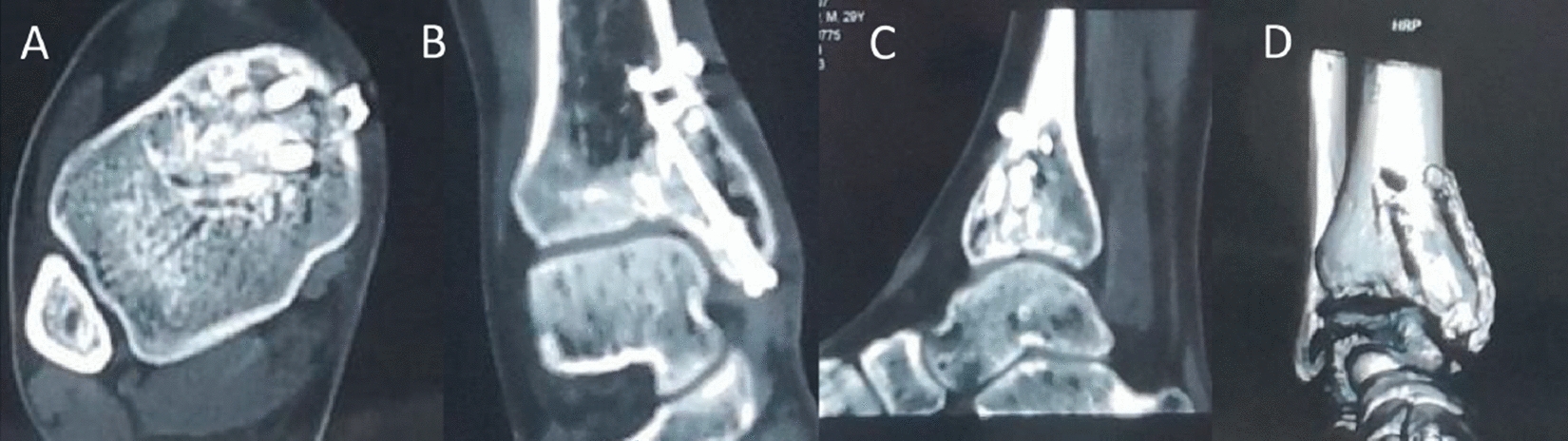
CT 1 year review of the ankle joint after surgery (**a**–**d**): resection of the distal right tibia lesion after bone graft fusion with plate and screw internal fixation. The bone graft was filled and fused well. Steel plate and screw fixed firmly, no fracture, bone nonunion, etc

**Fig. 10:**
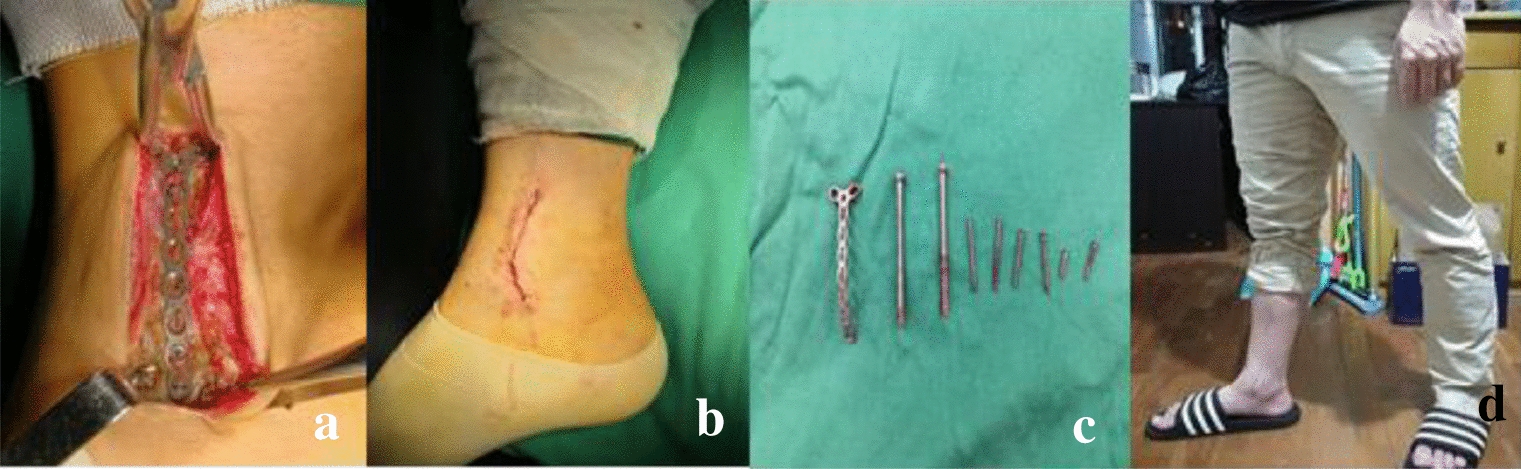
The fixation was removed within 2 years after the operation. Follow-up: the right distal tibia lesion was removed by bone graft fusion plate and screw internal fixation, and the plate and screw were removed completely (**a**–**c**). Good bone graft fusion, good ankle movement, no cardiopulmonary metastasis (**d**)

For case No. 12 (Figs. 3, 4, 5, 6, 7, 8, 9 and 10), the patient, (male, 28 years old), was admitted to the hospital via the outpatient department as “GCT and pathological fracture of the distal right tibia”, mainly due to “pain and swelling of the right ankle caused by a sprain from falling down some stairs, accompanied by limited movement for more than 2 months”. Received a diagnosis of HIV 3 years ago, on long-term oral highly active antiretroviral therapy (HAART), hepatitis C virus (HCV)-positive, syphilis antibody-positive, given penicillin treatment in their local hospital.

Physical examination: the right distal shank anterior tibial skin color was normal; no vein inflammation; skin temperature was slightly elevated; local tenderness; no obvious radiation pain; the right distal tibia could touch the rough bone surface; the right distal tibia axial rattling pain was positive; the right ankle joint flexion and extension activity was limited; the right ankle dorsiflexion was 10 degrees, plantar flexion 30 degrees, and varus 20 degrees; the left ankle had dorsiflexion of 25 degrees and plantar flexion of 50 degrees. The dorsal foot artery had a strong pulse, and the toes felt like blood transport was normal. Auxiliary test: viral load, target not detected; CD4 T lymphocytes, 375 cells/µl.

An X-ray of the ankle joint showed rounded, cystic destruction of the distal right tibia with expansion and thinning of the bone cortex (Fig. 3). CT results showed that distal round cystic bone destruction of the right tibia, expansions, internal trabeculae disruption, bone cortical rupture, and swelling of the surrounding soft tissue (Fig. 4). An MRI plain scan of the ankle joint (October 20, 2017) showed a mass at the distal end of the right tibia, about 29 × 13 × 24 mm, an abnormal constant signal, TIWL showed an equisignal with clear margin and visible bone destruction, these findings are considered to be diagnostic of giant cell tumor of bone (Fig. 5).

Tumor curettage using an ultrasonic knife, 95% alcohol, local chemotherapy with methotrexate, bone graft fusion, and internal fixation with a mini-plate and screw were performed (Fig. 6).

Hematoxylin and eosin (HE) staining (20×, 40×): A large number of giant cells and stromal cells was observed, accompanied by a small amount of focal hemorrhage and fibrous tissue hyperplasia. Immunohistochemical staining (20×, 40×): CD68 antigen-positive (Fig. 7).

Postoperative anteroposterior and lateral X-ray examination of the ankle joint at 1 week, 1 year, and 2 years: postoperative resection of the distal right tibia lesion with bone graft fusion-plate postoperative screw fixation. The bone graft was filled and fused well. The steel plate and screw were firmly fixed, with no fracture, bone nonunion, etc. (Fig. 8). CT 1 year review of the ankle joint after surgery: resection of the distal right tibia lesion after bone graft fusion with plate and screw internal fixation. The bone graft was filled and had fused well. The steel plate and screw were firmly fixed, with no fracture, bone nonunion, etc. (Fig. 9).

The fixation was removed within 2 years following the operation. Follow-up: the right distal tibia lesion was removed by bone graft fusion plate and screw internal fixation, and the plate and screw were completely removed. Good bone graft fusion, good ankle movement, and no cardiopulmonary metastasis were observed (Fig. 10).

## Discussion

HIV infection leads to severe B cell dysfunction, manifesting as an impaired humoral immune response to infection and vaccination, which cannot be completely reversed by other effective antiretroviral therapies (ART). Bone loss and osteoporosis lead to an increased incidence of brittle fracture, which in recent years has become an important non-AIDS comorbidity in patients with chronic HIV infection [14–16]. Interestingly, ART can exacerbate bone loss, especially in the first few years after starting treatment [17–19].

The mechanism of bone loss induced by HIV is multifactorial and is complicated by the association of HIV infection with multiple risk factors for osteoporosis and fracture, but the role B cells play in bone loss induced by HIV has only recently emerged. Although B cells are best known for their important antibody-producing ability, they also produce two cytokines that are critical to bone metabolism: the key osteoclast receptor activator NF-KB ligand (RANKL) and its physiological inhibitor osteoprotectin (OPG). Abnormal B cell production of OPG and RANKL has been shown to be a major factor in increasing the risk of bone loss and fracture in animal models and in HIV affected persons [20–23].

The maintenance of bone integrity requires the balanced regulation of bone remodeling processes, and tumor cells can disrupt this balance of bone remodeling and destroy the normal equilibrium, leading to various osteolytic or osteoblastic osteopathies. As mentioned earlier, GCT is a primary osteolytic tumor characterized by bone destruction. It consists of osteoclast-like giant cells, stromal cells, and monocytes. Stromal cells are considered to be tumor factors for GCT because they maintain proliferation in culture and are positive for the proliferation marker Ki67 [21, 24]. The expression of RANKL mRNA has been detected in GCT using a variety of techniques, such as RT-PCR, in situ hybridization, and immunofluorescence staining [25–27]. The proportion of stromal cells positive for RANKL/OPG was significantly increased in GCT samples compared with the proportion in non-osteolytic bone tumors, while the addition of exogenous OPG to cultured GCT cells inhibited bone resorption and osteoclast formation.

It has been reported that the toxic and side effects of HAART drugs can lead to the disorder of amino acid metabolism, which directly affects osteoblasts and osteoclasts, changes the homeostasis of bone structure, leads to mitochondrial dysfunction, aggravates the inflammatory process, and ultimately can lead to bone loss [28, 29]. Multiple studies have shown that among antiretroviral drugs tenofovir disoproxil fumarate (TDF) has the most severe effect on bone loss, causing a loss of about 3% to 6% of bone density during the first year of treatment, which increases with time [30]. The main reason is that TDF leads to downregulated gene expression and dysfunction in osteoblasts, resulting in reduced bone formation and decreased bone density. The activity of osteoblasts decreases with prolonged drug treatment. At the right, the probability of fragility fracture increases with the duration of retrotherapy. In the present study, all patients on ART in the HIV group were on TDF. Therefore, we speculated that the mechanism of HIV complicated with GCT may be related to a decline in normal host immunity, long-term chronic inflammation, T cell loss, monocyte/macrophage activation, RANKL overexpression, HIV toxemia, and the side effects of HAART.

The questions we were interested in was: How are these patients treated with surgical intervention? However, at present, there have been few reports about HIV-positive patients with GCT who have a pathological fracture, and there are currently no effective treatments for this disease.

All patients in the HIV group received HAART, their average CD4 count at the time of surgery was 434.9 absolute/mL, and the viral load control was excellent, with an average of 3758 copies/ml. There was no tumor recurrence, distant metastasis, or surgical site infection in any of these patients. In the HIV group, one case of giant cell tumor of the proximal tibia showed mild articular surface collapse and mild valgus deformity of the knee joint, but retained joint function. The MSTS scores of excellent and good in both the HIV and the control group was 83.3%, and thus there was no statistically significant difference (P > 0.05). Compared with their preoperative scores, the MSTS scores of the HIV group were significantly improved postoperatively, ranging from 7 to 11 points preoperatively to 24 to 27 points postoperatively; this difference was statistically significant (P < 0.05). Blackley et al. [31] reported that 59 cases of GCT were treated with high-speed grinding drill-assisted curettage, with an average follow-up period of 80 months and a recurrence rate of 12%. The postoperative recurrence rate of patients in our study was low, which may be related to the routine use of an ultrasonic scalpel during the operation. Using an ultrasonic scalpel can reduce the difficulty of surgery, decrease the operation time, remove bone crest tumor cells that cannot be scraped by conventional curettes, assist with edge resection, and ensure the thoroughness of the surgery. There is less tissue damage, making surgery safer, and the cutting temperature is low, at between 70 and 80 ℃, which is still sufficient to destroy any cancer cells, further reducing the likelihood of tumor recurrence.

Currently, the miniature locking bone plate used makes the fixation system better as it has a good fit, high plasticity, allows easy anatomical fixation, does not take up too much space, and stabilizes the fracture end to promote fracture healing. In our cases, a miniature locking bone plate was used to bridge the lesion bone defect area to provide sufficient fixation strength and promote the normal healing of fractures. There was no loosening or displacement of the internal fixator, and it was relatively easy to operate the technology of the bridging locking plate on either side of the fracture end after the removal of the lesion and bone grafting.

This study had some limitations. First, the sample size was small and the follow-up time was short, so the clinical curative effect of our approach remains to be further studied. Second, the MSTS93 scoring system can provide an evaluation scale for patients following limb surgery, but this system can be affected by subjective factors, including the degree of patient cooperation, a patient’s cognitive ability, and the physician’s clinical experience, thus it is difficult to achieve objective evaluation and the long-term efficacy remains to be seen.

## Conclusions

The use of an ultrasonic scalpel combined with a miniature locking bone plate and autogenous bone graft treatment showed satisfactory results in HIV-positive patients with giant cell tumor of long bone. During the surgical procedure, however, the nature, scope, and fracture damage of the lesion should be fully considered. During the operation, the lesion should be thoroughly scraped, the tumor wall should be polished using an ultrasonic scalpel, and the bone grafting should be put under pressure. At the same time, appropriate miniature locking bone-plate fixation is helpful for fracture healing.

## Data Availability

All data generated or analyzed during this study are included in this published article and its supplementary information files.

## Abbreviations

HIV: Human immunodeficiency virus
MSTS: Musculoskeletal Tumor society
GCT: Giant cell tumor of bone enzyme-linked
ELISA: Immunosorbent assay
CT: Computed tomography
MRI: Magnetic resonance imaging
HAART: Highly active antiretroviral therapy
